# Relationship between plasma Atherogenic index and final pathology of Bosniak III-IV renal masses: a retrospective, single-center study

**DOI:** 10.1186/s12894-019-0514-0

**Published:** 2019-09-13

**Authors:** Emre Karabay, Nejdet Karsiyakali, Serdar Duvar, Cagatay Tosun, Ahmet Ruknettin Aslan, Omer Ergin Yucebas

**Affiliations:** 1 Department of Urology, Haydarpasa Numune Training and Research Hospital, University of Health Sciences, Tibbiye Street. No: 23 34668 Uskudar /, ISTANBUL, Turkey; 2 Department of Urology, Cukurca State Hospital, Cukurca Devlet Hastanesi, Uroloji Klinigi, Cukurca/, HAKKARI, Turkey

**Keywords:** Atherogenic index of plasma (AIP), Benign, Carcinoma, Renal cell carcinoma

## Abstract

**Background:**

There is an increased incidence of renal cell carcinoma (RCC) in patients with metabolic syndrome who usually have high levels of serum triglyceride (TG) and low high-density lipoprotein-cholesterol (HDL-C). Plasma atherogenic index (PAI) is the logarithmic ratio of serum TG level to HDL-C and related to cardiovascular diseases. In this study, we aimed to determine the accuracy of PAI in determining renal malignancy in localized renal masses preoperatively.

**Methods:**

Totally 169 patients who were diagnosed with Bosniak III-IV lesions by imaging modalities and treated in our hospital with partial or radical nephrectomy were retrospectively analyzed using institutional renal cancer database between 2013 and 2018. Preoperative images were evaluated by two experienced radiologists. The patients were divided into two groups according to their postoperative pathological diagnosis as malignant or benign tumors. The PAI of each patient was calculated and the statistical significance of PAI in predicting malignancy for renal masses was analyzed using uni- and multivariable analyses.

**Results:**

Of patients, 109 (64.5%) were males and 60 (35.5%) were females with a median age of 61 (33–84) years. Median tumor size was 6.5 (2–18) cm. Pathological diagnosis was malignant in 145 (85.8%) and benign in 24 (14.2%) patients. There was no statistically significant difference in serum TG levels between malignant and benign cases (*p* > 0.05). The HDL-C levels were significantly lower in malignant cases (*p* = 0.001). Median PAI value was 0.63 (0.34–1.58) and significantly higher in malignant cases (*p* = 0.003). The PAI cut-off value for malignancy was ≥0.34. The sensitivity was calculated as 88.2% and specificity as 45.8%, the positive predictive value as 90.8, negative predictive value as 39.3, and odds ratio as 6.37 (95% CI: 2.466–16.458). In multivariable analysis, gender, smoking status, and hypertension had no effect on malignancy, whereas PAI and HDL-C were independent risk factors (*p* = 0.003 and *p* = 0.003, respectively). The risk of malignancy was 5.019 times higher, when PAI was > 0.34 (95% CI: 1.744–14.445) in multivariable logistic regression analysis.

**Conclusions:**

The PAI can be used as a predictive tool in suspicion of malignant renal masses. In case of a benign pathology, PAI levels may be encouraging for surgeons for nephron-sparing surgery.

## Background

Renal cell carcinoma (RCC) is 2 to 4% of all newly diagnosed tumors in adults [1]. Renal masses are often found incidentally in ultrasonography (USG), computed tomography (CT), and magnetic resonance imaging (MRI) which are performed to evaluate other pathologies [2]. These are called localized renal masses and, although most of them are malignant in pathological examination, about 20 to 30% of specimens are still diagnosed with benign lesions [3]. Tumor biopsy is the only histological diagnostic method in the preoperative period, particularly in small renal masses. In addition, CT imaging is the standard tool to detec renal masses; however, it is not reliable in distinguishing different histological types of tumors [4]. Also, MRI and USG are helpful in evaluating complex renal masses, although they have some limitations [5, 6]. In a systematic review, overall median diagnostic sensitivity and specificity in predicting RCC were reported as 88% (IQR 81–94%) and as 75% (IQR 51–90%), respectively for CT and as 87.5% (IQR 75.25–100%) and as 89% (IQR 75–96%), respectively [7].

Well-defined risk factors for RCC include tobacco use, obesity, and hypertension (HT). Also, hyperglycemia, hypertrigliseridemia, low high-density lipoprotein HDL cholesterol (HDL-C) levels, and metabolic syndrome with obesity have been shown to be related with RCC [8]. Obesity is a known risk factor for renal cancer. Although lipid peroxidation and oxidative stress have been primarily blamed, the exact underlying biological mechanisms are still unclear [9, 10]. In addition, dyslipidemia has been shown to increase oxidative stress and chronic inflammation and to be associated with insulin resistance and metabolic syndrome [11]. Lipids which are the major cell membrane components promote cell growth and alterations in their concentrations may occur during carcinogenesis [12]. In general, RCC patients with hypertrigliseridemia have a lower progression-free and cancer-specific survival rates [13]. High triglyceride (TG)/HDL-C ratio indicates an atherogenic lipid profile, which is an independent risk factor for cardiovascular diseases [14, 15]. In recent studies, the Plasma Atherogenic Index (PAI) is used as a reliable indicator for cardiovascular diseases [16, 17] and has been shown to be associated with metabolic syndrome and high body weight [18, 19]. The PAI is the logarithmic ratio of the plasma TG to HDL-C levels [log (TG / HDL-C)] [20]. It is a simple calculation without an extra cost.

In the present study, we aimed to determine the accuracy of PAI in determining renal malignancy in localized renal masses preoperatively.

## Methods

We retrospectively reviewed the medical records of patients with renal mass who underwent partial or radical nephrectomy in our hospital between the years 2013 and 2018. Approval was obtained from the institutional Ethics Committee. Patients were selected based on the existence of Bosniak III or IV lesions as evidenced by imaging modalities. Patients with incomplete data for lipid profiles before the operation, missing pre-operative diagnostic images, and those who were already on lipid-lowering agents, such as statin and fenofibrate, were excluded from the study. Metabolic syndrome was not considered an exclusion criterion. Although smoking may disrupt the lipid profile, smoking is a well-defined risk factor for RCC; therefore, it was not considered an exclusion criterion, either. A total of 169 patients who met the inclusion criteria and were randomly selected were included in the study. Two experienced radiologists evaluated preoperative images of the patients. Another experienced radiologist evaluated the images, when the diagnosis of malignancy was suspected. Two experienced uropathologists evaluated postoperative specimens of the patients. The patients were divided into two groups according to their pathological diagnosis as malignant or benign renal tumors. The PAI of each patient was calculated using the formula log [plasma TG level / plasma HDL-C level]. Multivariable logistic regression analysis was used to analyze the predictive factors for malignancy. The role of PAI in predicting malignancy of renal masses was evaluated.

A written informed consent was obtained from each patient. The study was conducted in accordance with the principles of the Declaration of Helsinki.

### Statistical analysis

Statistical analysis was performed using the Number Cruncher Statistical System (NCSS) version 2007 software (NCSS LLC., Kaysville, UT, USA). Descriptive data were expressed in mean and standard deviation (SD), median (min-max), or number and frequency. Normal distribution of the quantitative data was analyzed using the Shapiro-Wilk test. The Student’s t-test was used to compare two groups of quantitative variables showing normal distribution and Mann-Whitney U test was used to compare two groups of quantitative variables which did not show normal distribution. The analysis of variance (ANOVA) test was used in comparison of more than two groups of quantitative variables showing normal distribution. The Kruskal-Wallis test was used to compare more than two groups of quantitative variables which did not show normal distribution. The Pearson chi-square test was used to compare the qualitative data. Diagnostic scanning tests including sensitivity, specificity, positive predictive value (PPV), negative predictive value (NPV), and Receiver Operating Characteristics (ROC) analysis were carried out to determine the predictive value for the parameters. The correlation between age, gender, side, size, and localization of the tumor, smoking status, diabetes mellitus (DM), HT, body mass index (BMI), HDL-C, TG, and PAI and malignancy were evaluated in univariable analyses. The multivariable logistic regression analysis was used in four models to investigate the effect of HDL-C, PAI, and TG on malignancy. In all models, gender, BMI, smoking status, and HT were independent variables, while HDL-C in the first model, PAI in the second model, TG in the third model, and PAI in the fourth model was additionally included. In these analyses, the impact of PAI on malignancy was evaluated alone and combined with HDL-C and TG. The DeLong method of area under curve (AUC) of the ROC curve was conducted to analyze significant differences. A *p* value of < 0.05 was considered statistically significant.

## Results

A total of 169 patients, 109 (64%) were males and 60 (36%) were females with a median age of 61 (33–84) years. Pathological diagnosis was a malignant renal lesion in 145 cases (85.8%) and a benign renal lesion in 24 cases (14.2%). The median tumor size was 6.5 (2–18) cm. The mean BMI was 29.00 ± 4.28 kg/m^2^. The median value of PAI was 0.53 (0.15–1.58) (Table 1). Demographic and tumoral characteristics of the patients are summarized in Table 1.

**Table 1:** Demographics of Patients

Age (year)	median (min-max)	61 (33–84)
Gender (n, %)	Male	109 (64.5%)
	Female (n, %)	60 (35.5%)
Side (n, %)	Right (n, %)	84 (49.7%)
	Left (n, %)	85 (50.3%)
Body Mass Index (kg/m^2^)	mean ± SD	29.00 ± 4.28
Tumour Size (cm)	median (min-max)	6.5 (2–18)
Tumour Localisation (n, %)	Lower Pole	26 (15.4%)
	Middle Pole	97 (57.4%)
	Upper Pole	46 (27.2%)
Tumour Type (n, %)	Clear Cell	114 (67.5%)
	Chromophobe Cell	16 (9.5%)
	Papillary	15 (8.9%)
	Angiomyolipoma	11 (6.5%)
	Oncocytoma	13 (7.7%)
Malignancy (n, %)	Malign	145 (85.8%)
	Benign	24 (14.2%)
Fuhrman Grade (n, %)	Grade 2	43 (33.3%)
	Grade 3	71 (55.0%)
	Grade 4	15 (11.6%)
Tumour Stage (n, %)	T1a	24 (16.7%)
	T1b	36 (25.0%)
	T2a	16 (11.1%)
	T2b	6 (4.2%)
	T3a	46 (31.9%)
	T3b	11 (7.6%)
	T4	5 (3.5%)
Smoking Status (n, %)	Exist	116 (68.6%)
	None	53 (31.4%)
Diabetes Mellitus (n, %)	Exist	67 (39.6%)
	None	102 (60.4%)
Hypertension (n, %)	Exist	105 (62.1%)
	None	64 (37.9%)
Triglyceride Value [mg/dl]	median (min-max)	145 (68–1019)
HDL-Cholesterol Value [mg/dl]	mean ± SD	41.12 ± 11.94
Plasma Atherogenic Index Value	median (min-max)	0.53 (0.15–1.58)

There was no statistically significant relationship between malignancy and patient age, side, size, and localization of the tumor, and DM status of the patient. In male patients, malignancy rate was higher than females (*p* = 0.039). The BMI was significantly higher in malignant patients (*p* = 0.023). The number of smoker patients and those with HT was significantly higher in malignant group (*p* = 0.009 and *p* = 0.026, respectively). Plasma HDL-C levels were significantly lower in malignant cases (*p* = 0.001). There was no significant difference in plasma TG levels between malignant and benign cases (*p* > 0.05). The PAI of malignant cases was significantly higher than the PAI of benign cases (*p* = 0.003) (Table 2).

**Table 2:** Comparisons of Malignant and Benign Patients

		Malignancy		
		Malignant (*n* = 145, 85.8%)	Benign (*n* = 24, 14.2%)	*p*
Age (year)	median (min-max)	61 (33–84)	63 (39–76)	^a^0.472
Gender (n, %)	Male	98 (89.9)	11 (10.1)	^b^0.039^*^
	Female	47 (78.3)	13 (21.7)	
Side (n, %)	Right	73 (86.9)	11 (13.1)	^b^0.682
	Left	72 (84.7)	13 (15.3)	
Size (cm)	median (min-max)	8 (2–18)	5 (3–11)	^a^0.223
Localization (n, %)	Lower Pole	23 (88.5)	3 (11.5)	^b^0.848
	Middle Pole	82 (84.5)	15 (15.5)	
	Upper Pole	40 (87.0)	6 (13.0)	
Smoking Status (n, %)	Exist	105 (90.5)	11 (9.5)	^b^0.009^**^
	None	40 (75.5)	13 (24.5)	
Diabetes Mellitus (n, %)	Exist	57 (85.1%)	10 (14.9%)	^b^0.827
	None	88 (86.3%)	14 (13.7%)	
Hypertension (n, %)	Exist	95 (90.5%)	10 (9.5%)	^b^0.026^*^
	None	50 (78.1%)	14 (21.9%)	
Body Mass Index (kg/m^2^)	mean ± SD	29.30 ± 4.36	27.16 ± 3.27	^c^0.023^*^
Triglyceride Value [mg/dl]	median (min-max)	156 (68–1019)	164 (100–226)	^a^0.383
HDL-Cholesterole Value [mg/dl]	mean ± SD	39.64 ± 11.53	50.04 ± 10.59	^c^0.001^**^
Plasma Atherogenic Index Value	median (min-max)	0.63 (0.34–1.58)	0.62 (0.39–0.76	^a^0.003^**^

^*a*^*Mann Whitney U Test,* ^*b*^*Pearson Chi-Square Test,* ^*c*^*Student-t Test,* ^*^p < 0.05 ^**^p < 0.01

The ROC curve analysis showed that the cut-off value for malignancy was 0.34. The PAI cut-off value (0.34) had a sensitivity of 88.2% and a specificity of 45.8%. The PPV was 90.8 and NPV was 39.3. The AUC of the ROC curve was 69% and standard error was 5.8%. For the cut-off value of 0.34, the odds ratio was 6.37 (95% CI: 2.466–16.458).

Univariable analysis revealed that significant factors related to malignancy were gender, smoking status, HT, BMI, HDL-C level, and PAI. The effect of these variables was also evaluated by multivariable logistic regression analysis. According to the results of multivariable analyses, the effect of gender, smoking status, and HT on malignancy was not significant. A PAI of ≥0.34 increased the risk of malignancy by 5.019 folds (95% CI: 1.744–14.445) (Table 3 - Model 2). Similarly, low values of HDL-C (F < 55; M < 45) increased the risk of malignancy [5.019 folds (95% CI: 1.744–14.445) in Model 1 and 2.062 (95% CI: 0.738–5.767) in Model 4, respectively] (Table 3).

**Table 3:** Results of multivariable logistic regression analysis according to malignancy

Model		Odds Ratio (95%CI)	*p*	AuROC (95%CI)	*p*
1	Gender (M^a^)	0.764 (0.246–2.367)	0.640	0.772 (0.672–0.873)	< 0.001^*^
	BMI	1.135 (1.002–1.287)	0.047^*^		
	Smoking Status	1.887 (0.63–5.65)	0.257		
	HT	2.296 (0.874–6.03)	0.092		
	HDL-Cholesterole (F^b^ < 55; M^a^ < 45)	5.019 (1.744–14.445)	0.003^*^		
2	Gender (M^a^)	0.764 (0.246–2.367)	0.640	0.787 (0.687–0.887)	< 0.001^*^
	BMI	1.135 (1.002–1.287)	0.047^*^		
	Smoking Status	2.296 (0.874–6.03)	0.092		
	HT	1.887 (0.63–5.65)	0.257		
	Plasma Atherogenic Index (≥0.34)	5.019 (1.744–14.445)	0.003^*^		
3	Gender (M^a^)	0.572 (0.2–1.633)	0.296	0.760 (0.663–0.857)	< 0.001^*^
	BMI	1.124 (0.996–1.269)	0.059		
	Smoking Status	2.157 (0.767–6.068)	0.145		
	HT	2.278 (0.902–5.753)	0.081		
	TG	1.003 (0.995–1.011)	0.456		
4	Gender (M^a^)	0.516 (0.175–1.52)	0.230	0.773 (0.677–0.870)	< 0.001^*^
	BMI	1.13 (0.999–1.279)	0.052		
	Smoking Status	1.654 (0.538–5.084)	0.380		
	HT	2.181 (0.857–5.555)	0.102		
	HDL-Cholesterole (F^b^ < 55; M^a^ < 45)	2.062 (0.738–5.767)	0.168		
	TG	1.003 (0.995–1.011)	0.496		

^a^ Male, ^b^ Female, ^*^p < 0.05

**Fig. 1:**
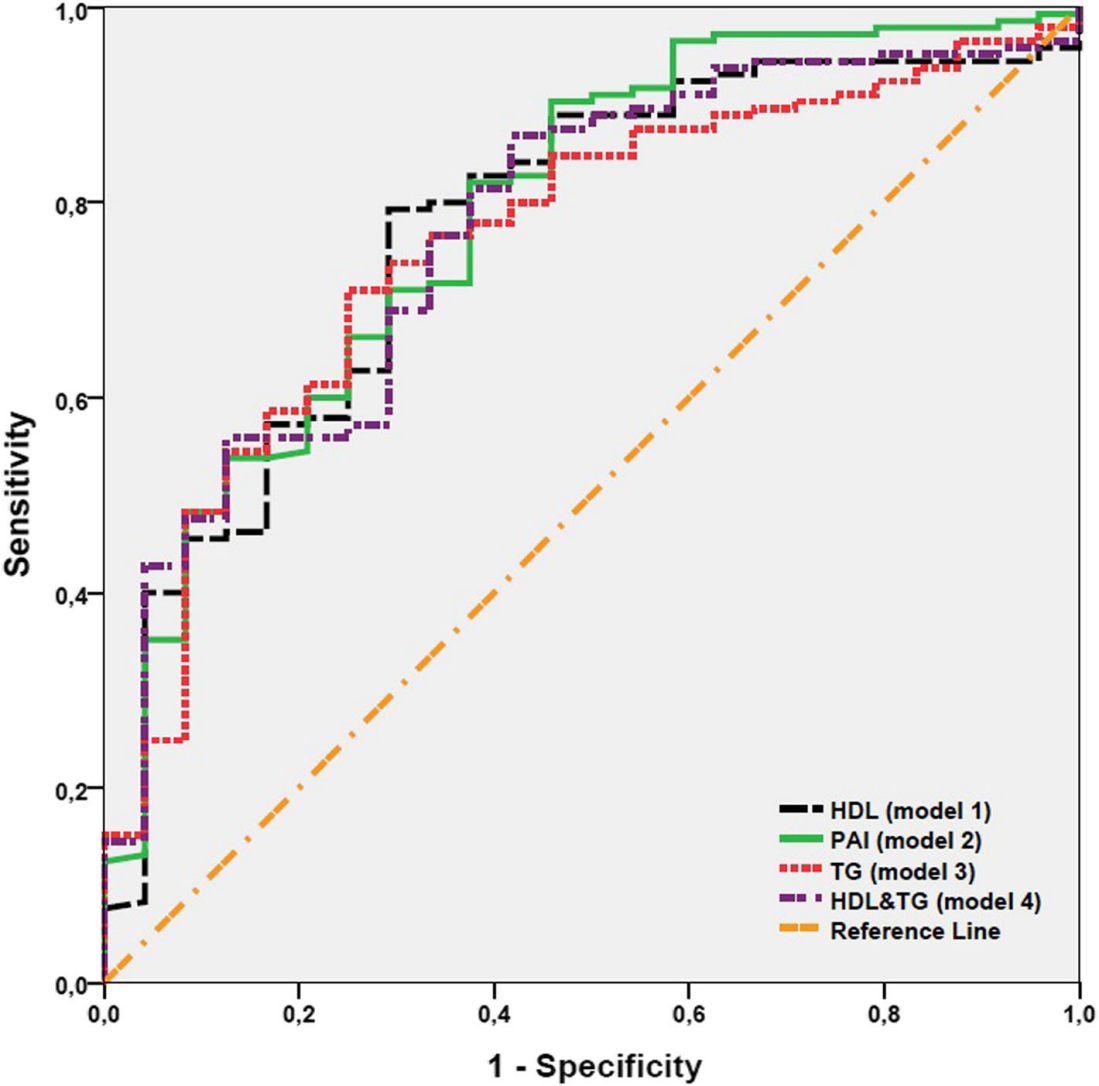
ROC Curve for Models According to Malignancy

All models predicting malignancy revealed a statistically significant outcomes (*p* < 0.001, for all). Although the AUC of the ROC curve showed no statistically significant difference (*p* > 0.05, for all), the highest AUC of the ROC curve was observed in Model 2 which included PAI (Table 3 & Fig. 1). No significant difference was found in PAI between the Fuhrman grades (*p* > 0.05).

## Discussion

In this retrospective study, we showed that PAI and low values HDL-C of the patients with pathologically proven malignant renal tumors were significantly higher, compared to PAI of patients with benign renal lesions. Based on the multivariable logistic regression analysis, the malignancy risk was 4.55 folds higher in the patients with a PAI of > 0.34.

Dobiasova et al. [20] were the first to publish about log (TG/HDL-C) formula which they named the PAI. They found that this ratio was directly proportional to fractional esterification of HDL-C, inversely proportional to the particle size of LDL-C, and there was a direct relation between the PAI and atherosclerosis risk. Later studies showed a correlation between PAI and acute coronary syndrome and other cardiovascular diseases [16, 17].

In an epidemiological study, Haggstrom et al. [21] reported that increased BMI, serum glucose, and TG levels were significant risk factors for renal cancer in male patients, while only increased BMI was found a significant risk factor in female patients. As the relationship between obesity and renal tumors is well-known and obesity has blamed for one of the underlying pathologies for impaired lipid metabolism, a number of studies have attempted to investigate the possible relationship between lipid levels and renal cancer. In the Metabolic Syndrome and Cancer project (Me-Can) examining the relationship between serum TG levels and cancer in patients with metabolic syndrome, a significant correlation was found between TG levels and renal cancer (*p* = 0.001 RR: 1.96 (1.51–2.46)) [22]. However, in another study conducted by the Vorarlberg Health Monitoring and Promotion Program (VHM&PP) Study Group, increased TG levels were not associated with an increased risk for cancer (*p* = 0.105 HR:1.27 (0.95–1.69)) [23]. In the Apolipoprotein-related Mortality Risk (AMORIS) study, there was a significant correlation between TG and renal cancer risk (*p* = 0.001) and there was a negative correlation between total cholesterol and HDL-C and renal cancer risk (*p* = 0.001 and *p* = 0.075, respectively) [24]. In our study, although gender, HT, and smoking which are well-known risk factors for renal caner were found to be significant risk factors in the univariable analysis, we found no significant relationship between TG levels and renal cancer (*p* > 0.05). Although previous studies have shown a correlation between lipid levels and renal cancer, there is no study available in the literature comparing patients with suspected malignancy as assessed by imaging studies and those with a benign pathological diagnosis. In the AMORIS study, there was a significant correlation of the TG/HDL-C ratio with renal cancer, but not with cancer [24]. In this large-scale, comprehensive study, the cut-off value of the log (TG/HDL-C) to prevent cardiovascular diseases was calculated similar to previous studies (0.5) However, in our study, we used a cut-off value of 0.34, which suggested that PA with other variables was a predictor of renal cancer. Despite small sample size in our study, the reason for the discrepancy in our results and AMORIS findings may be due to different cut-off values used. Nonetheless, the use of different cut-off values for cardiovascular diseases and renal cancer is reasonable, since the pathophysiological mechanisms of both diseases are different.

Furthermore, it is well-established that systemic inflammatory response has a negative effect on survival of RCC patients [25]. Vivar et al. [26] reported that inflammation has a significant, negative impact on the prognosis and progression of clear-cell RCC. Systemic inflammation induces some alterations in lipid metabolism [27]. Changes in TG metabolism also affect HDL-C metabolism [28]. These alterations also modify PAI. Since renal malignancy may present with systemic inflammation and lipid metabolism changes, it is reasonable to use PAI to predict malignant renal masses in the preoperative period.

In addition, PAI has been shown to be related with metabolic syndrome and high body weight [18, 19]. Metabolic syndrome characterized by hyperglycemia, hypertension, hypertriglyceridemia, low HDL-C levels, and abdominal obesity has a strong correlation with RCC [8]. Progression-free and cancer-specific survival in RCC is worse, when TG levels are high [13]. In our study, there was no statistically significant difference between TG levels of malignant and benign cases (*p* > 0.05). On the other hand, HDL-C levels of malignant cases were found to be significantly lower than benign cases (*p* = 0.001). Accordingly, PAI of malignant cases were significantly higher than benign cases (*p* = 0.003). Based on these findings, we believe that, in patients with suspected renal masses on their preoperative radiological images, PAI can be used as an additional predictive tool of malignancy and be helpful for planning the type of the operation (partial or radical nephrectomy).

### Limitations

Nonetheless, there are some limitations to this study. First, it was a retrospective, observational study and the inherent retrospective and non-randomized nature might have led to selection bias. Second, only patients of a single center were included and the sample size was small. Third, we evaluated only patients who underwent surgery for renal masses and we were unable to compare symptomatic and incidental cases. These conditions might have also led to selection bias and may be not representative of general population. Nevertheless, our study showed a direct correlation between PAI and malignant renal masses which should be investigated in further large-scale, prospective, multi-center studies.

## Conclusions

In conclusion, the incidence of newly diagnosed localized renal masses has been increasing with the routine use of advanced imaging modalities. Our study results suggest that PAI can be used as a predictive tool preoperatively to distinguish benign cases from malignant ones and be encouraging for the surgeons to consider performing nephron-sparing surgery. However, further large-scale, prospective, and multi-center studies are needed to establish a definite conclusion.

## Data Availability

All the data supporting our findings is contained in the manuscript. The datasets used and/or analysed in the current study is available from the corresponding author on reasonable request.

## Abbreviations

CT: Computed tomography
HDL-C: High-density lipoprotein cholesterol
MRI: Magnetic resonance imaging
MS: Metabolic syndrome
PAI: Plasma atherogenic index
RCC: Renal cell carcinoma
TG: Triglyceride
USG: Ultrasonography
